# Insulin-like growth factor 2 mRNA-binding protein 1 (IGF2BP1) in cancer

**DOI:** 10.1186/s13045-018-0628-y

**Published:** 2018-06-28

**Authors:** Xinwei Huang, Hong Zhang, Xiaoran Guo, Zongxin Zhu, Haibo Cai, Xiangyang Kong

**Affiliations:** 1 Faculty of Environmental Science and Engineering, Kunming University of Science and Technology, Kunming City, 650504 Yunnan Province China; 2 Medical School, Kunming University of Science and Technology, Kunming City, 650504 Yunnan Province China; 3 Department of Rehabilitation Medicine, The First Affiliated Hospital of Chengdu Medical College, Chengdu City, 610500 Sichuan Province China; 4 Department of Oncology, Yunfeng Hospital, Xuanwei City, 655400 Yunnan Province China

**Keywords:** IGF2BP, IGF2 mRNA-binding protein, IMP, CRD-BP, VICKZ, Cancer

## Abstract

The insulin-like growth factor-2 mRNA-binding protein 1 (IGF2BP1) plays essential roles in embryogenesis and carcinogenesis. IGF2BP1 serves as a post-transcriptional fine-tuner regulating the expression of some essential mRNA targets required for the control of tumor cell proliferation and growth, invasion, and chemo-resistance, associating with a poor overall survival and metastasis in various types of human cancers. Therefore, IGF2BP1 has been traditionally regarded as an oncogene and potential therapeutic target for cancers. Nevertheless, a few studies have also demonstrated its tumor-suppressive role. However, the details about the contradictory functions of IGF2BP1 are unclear. The growing numbers of microRNAs (miRNAs) and long non-coding RNAs (lncRNAs) have been identified as its direct regulators, during tumor cell proliferation, growth, and invasion in multiple cancers. Thus, the mechanisms of post-transcriptional modulation of gene expression mediated by IGF2BP1, miRNAs, and lncRNAs in determining the fate of the development of tissues and organs, as well as tumorigenesis, need to be elucidated. In this review, we summarized the tissue distribution, expression, and roles of IGF2BP1 in embryogenesis and tumorigenesis, and focused on modulation of the interconnectivity between IGF2BP1 and its targeted mRNAs or non-coding RNAs (ncRNAs). The potential use of inhibitors of IGF2BP1 and its related pathways in cancer therapy was also discussed.

## Background

**Fig. 1:**
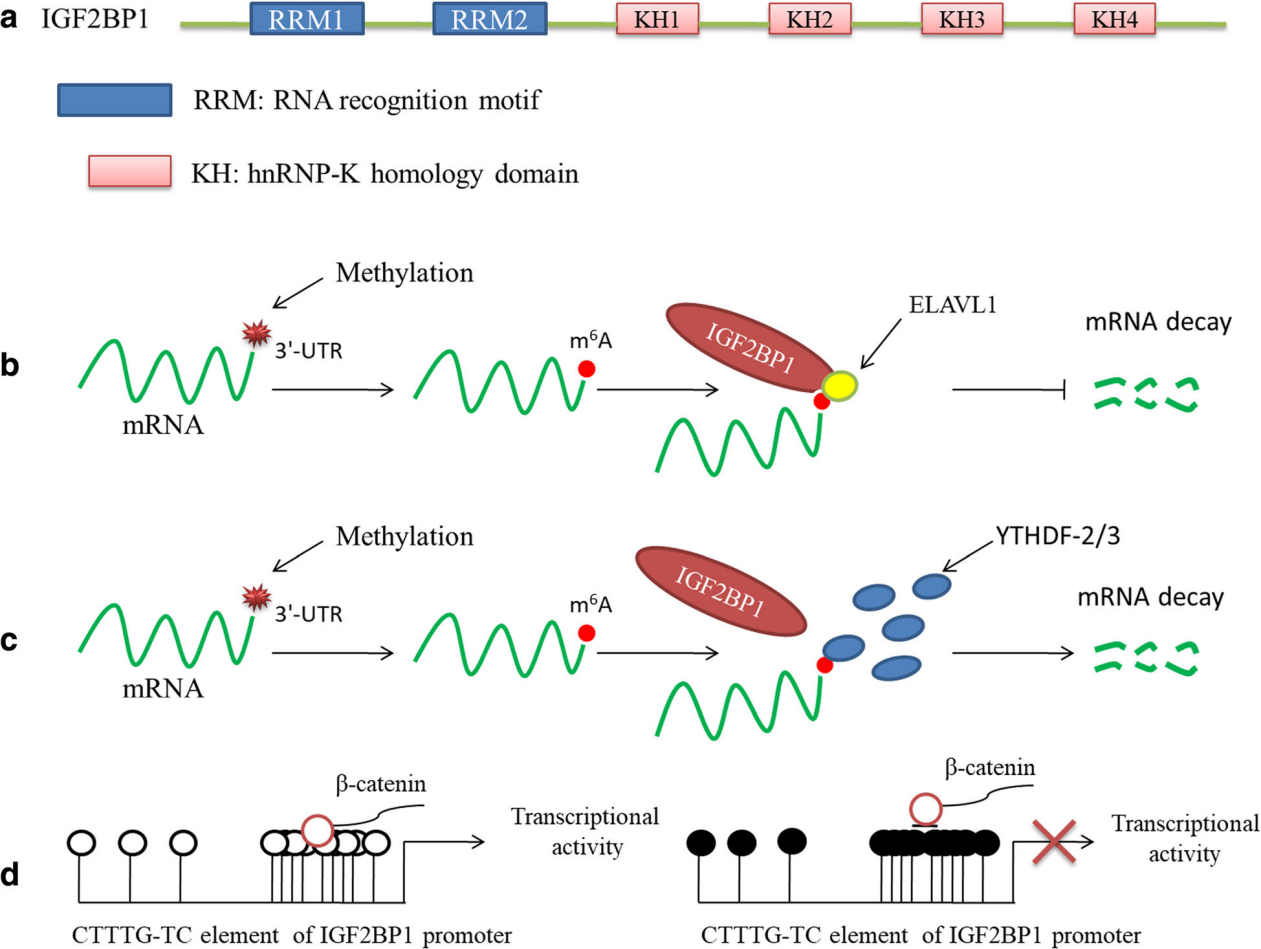
The KH domains of IGF2BP1 recognize and bind m^6^A-mRNAs, as well as the potential fate selection of IGF2BP1-targeted mRNAs. **a** Domain structure of human IGF2BP1. RNA-binding domains include two RNA recognition motifs (RRMs, blue) and four hnRNP-K homology domains (KH, red) [5]. **b** Schematic structures showing that mRNAs are methylated at the 3′-UTR by methyltransferase complex then recognized by IGF2BP1 under the co-effects of stabilizers such as ELAVL1, which finally inhibits the decay of m^6^A-RNAs [10]. **c** YT521-B homology (YTH) domain-containing proteins (YTHDFs) compete for the same m^6^A sites with IGF2BP1 and promote decay of m^6^A-RNAs. **d** The β-catenin physically binds to the element (CTTTG-TC) located in the promoter of IGF2BP1, which contributes to IGF2BP1 transcription activity (left) [12–15]. The hypermethylation of element (CTTTG-TC) blocks β-catenin binding to the region and thus suppresses IGF2BP1 transcription activity (right), leading to increased proliferation and migration of metastatic breast cancer cells [54, 103]

The insulin-like growth factor-2 mRNA-binding protein 1 (IGF2BP1), a member of a conserved family of single-stranded RNA-binding proteins (IGF2BP1-3), expresses in a broad range of fetal tissues and more than 16 cancers but only in a limited number of normal adult tissues. This gene is required for the transport of certain mRNAs that play essential roles in embryogenesis, carcinogenesis, and chemo-resistance [1, 2], by affecting their stability, translatability, or localization [1, 3, 4]. IGF2BP1 consists of six canonical RNA-binding domains, including four K homology (KH) domains and two RNA recognition motifs (RRMs) (Fig. 1a) [5]. Even though the RRM domains of IGF2BP1 can potentially contribute to the stabilization of IGF2BP-RNA complexes in a target-dependent manner [6], in vitro studies indicated that RNA binding was majorly facilitated by the KH domains [7]. The KH1/2 domain is significant for the stabilization of IGF2BP-RNA complexes. For example, the KH1/2 domain could regulate binding of IGF2BP1 to cis-determinants in the ACTB 3′-UTR as well as, more strikingly, the MYC-CRD (coding region stability determinant) RNA in vitro [8]. However, recent structural analyses of KH3/4 domain of human IGF2BP1 showed the formation of an antiparallel pseudo-dimer conformation where KH3 and KH4 each contacts the targeted RNA [9]. As far as we know, the KH domains, particularly the KH3–4 di-domain, are essential for the binding of IGF2BP1 to targeted mRNAs in a N6-methyladenosine (m^6^A)-dependent manner in which KH domains recognize the consensus GG (m^6^A) C sequence of mRNAs [10]. IGF2BP1 is considered as a m^6^A-binding protein, with > 3000 mRNA transcript targets [11]. Importantly, as shown in Fig. 1b, the m^6^A alterations of those mRNAs are required for the targeting of IGF2BP1 with mRNAs such as MYC, as well as for IGF2BP1-mediated control of mRNA expression. In addition, some co-factors of IGF2BP1, such as ELAV-like RNA binding protein 1 (ELAVL1), which may be recruited by IGF2BP1, protect m^6^A-containing mRNAs from degradation and subsequently promote their translation [10]. However, other m^6^A-binding proteins may be also the readers of the m^6^A-containing mRNAs that are also recognized by IGF2BP1, whereas result in different effects on those mRNAs from that of IGF2BP1 does. A set of YT521-B homology (YTH) domain-containing proteins (YTHDFs), for instance, can read and control the fate of m^6^A-containing mRNAs via modulating pre-mRNA splicing, promoting translation, or facilitating mRNA decay (Fig. 1c) [12–15].

In numerous studies in vivo and in vitro, the emerging cancer-related mRNAs have been found, including PTEN, ACTB, MAPK4, MKI67, c-MYC, and CD44. By regulating those mRNAs, IGF2BP1 has been identified to play important roles in cell proliferation and growth of normal tissues and tumor tissues, as well as tumor cell adhesion, apoptosis, migration, and invasion [8]. Thus, IGF2BP1 is considered to be one of the most promising therapeutic targets for treating cancers, as well as the use of inhibitors of IGF2BP1-mediated cell signaling would emerge as a potential strategy for cancer treatment. However, a few recent studies have found its suppressive role in tumor growth and metastasis [16, 17]. Therefore, mechanisms resulting in the paradoxical findings need to be elucidated. Additionally, the emerging non-coding RNAs (ncRNAs) including microRNAs (miRNAs) and long non-coding RNAs (lncRNAs) have been demonstrated to be involved in the mediation of cancer onset and progression by targeting IGFBP1 and thus could become novel therapeutic targets of cancers. In this review, we provided a new overview of the roles of IGF2BP1 in embryo development and in multiple cancers. We focused on the interconnectivity between IGF2BP1 and its targeted mRNAs or ncRNAs involved in the biological processes of embryogenesis and tumorigenesis, as well as aid in the identification of potential targets for cancer therapy and contribute to the cancer drug-discovery research.

## IGF2BP1’s role in modulating embryogenesis

The essential role of IGF2BP1 in embryo development has been established. IGF2BP1 is highly expressed during the stages between zygote and embryo phases, and nearly abolished in the normal adult organism [8]. In embryo studies of Xenopus, zebrafish, and mice, this gene was found to be expressed in various developmental cell types, including the migrating neural crest and branchial arches, and cranial neural crest (CNC) [18]. Notably, its modest expression was observed in the lung, spleen, and brain of 16-week-old male mice [8]. However, one report found that IGF2BP1 is not only ubiquitously expressed in organs during human embryonic development, but also much less presented in adult human prostate, testis, kidneys, and ovaries [19]. Therefore, the expression pattern observed for IGF2BP1 could indeed be characterized as “oncofetal,” since it is largely absent from normal adult tissues.

Mice with IGF2BP1 deficiency exhibit dwarfism, severely decreased viability, and impaired gut development. Similarly, knockdown of this gene may lead to 60% of perinatal death and significantly smaller body with hypoplastic tissues among almost all organs in mice [20]. The SNP rs9674544 in IGF2BP1 was identified to be significantly associated with primary tooth development in infancy [21]. Furthermore, in the neuronal development, IGF2BP1 regulates the neurite outgrowth, neuronal cell migration, and axonal guidance partially by controlling the spatiotemporal activation of protein synthesis such as ACTB mRNA [22, 23]. IGF2BP1 controls the subcellular sorting of the ACTB in primary fibroblasts and neurons by binding to the cis-acting zipcode in the ACTB’s 3-UTR [24]. Moreover, IGF2BP1 was also involved in determining cell fate in testis stem cells and controlling neuronal differentiation and matured neuronal system during regeneration [25–27]. One study showed that downregulation of IGF2BP1 expression in the dorsal neural tube was both necessary and sufficient for the delamination and emigration of CNC, whereas inhibition of its expression enhanced CNC delamination and induced epithelial dissociation. Furthermore, IGF2BP1 expression is negatively associated with epithelial-to-mesenchymal transition and plays an important role in sustaining epithelial integrity. Those regulation processes might involve partly the mechanism of which IGF2BP1 interacts with ITGA6 mRNA, either directly or indirectly, to control its expression [28]. In another study on the role of IGF2BP1 in amphibian neural crest migration, the reduced IGF2BP1 expression by antisense morpholino oligonucleotides (AMO), throughout the entire embryo, was showed to increase CNC migration, suggesting the reduction in CNC migration originally observed in the AMO-injected Xenopus embryos is a result of a global, non-cell autonomous reduction in IGF2BP1 expression [18]. Those findings reveal the essential roles of IGF2BP1 in regulating cell growth and differentiation during development of organisms and suggest that aberrant expression of this gene could cause dysplasia of tissues and organs by dysregulating the expression levels of its targets such as ACTB mRNA and ITGA6 mRNA.

So far, the regulatory networks that IGF2BP1 participates in embryonic development are little known. Interestingly, a novel ultra-conserved lncRNA, THOR (ENSG00000226856), which is testis-specific in adult tissues of human, zebrafish, and mouse, has been demonstrated to be broadly expressed during the early development of both zebrafish and mouse. By IGF2BP1 binding to exons 2 and 3 of THOR, it regulates IGF2BP1’s target mRNA levels to promote tumorigenesis [29]. Therefore, it may be deduced that THOR involves the development of organs and tissues and tumorigenesis by modulating IGF2BP1 expression levels, but the specifically regulated target mRNAs remain to be explored.

## Aberrant expression and the roles of IGF2BP1 in cancers

IGF2BP1 and 3 have an amino acid sequence identity of 73% with each other and many the same or similar functions in cytosol. The main IGF2BP family member described in the context of cancers is IGF2BP3 [30, 31]. The comprehensive description in the regulative mechanisms of IGF2BP1 in human cancers is little, although this gene has been demonstrated to play important roles in tumorigenesis and drug-resistance of cancer therapy in vitro and in vivo studies. Even though most of the presently found cancer-related mRNA targets of IGF2BP1 have been identified to promote tumor proliferation and growth, migration, and invasion, some mRNAs have been indicated to at least indirectly suppress tumor growth and metastasis. Additionally, some ncRNAs were found to involve the regulation of tumor events by targeting IGF2BP1. Thus, bearing in mind the described limitation of post-transcriptional modulation of gene expression mediated by RNA-binding proteins (RBPs), miRNAs, and lncRNAs in determining fate of tumorigenesis, we in the following review recent findings on the expression of IGF2BP1 in cancers and focus on the interconnectivity of IGF2BP1 with its targeted mRNAs or ncRNAs.

### Lung and esophageal cancer

The in vitro and in vivo studies, and TCGA data indicated that IGF2BP1 is significantly overexpressed in non-small cell lung cancer (NSCLC), especially both lung squamous cell carcinoma (LUSC) and lung adenocarcinoma (LUAD), and that its high expression correlates with the disease progression [32, 33]. IGF2BP1 overexpression was showed to significantly associate with younger onset age in LUSC and bigger tumor size and poor overall survival in LUAD [32]. Notably, another analysis from TCGA data indicated that the higher expression of IGF2BP1 is associated with poorer survival in esophageal adenocarcinomas (EAC), LUAD, and the pooled several other adenocarcinomas (ADCs) including cancers of the endometrium, prostate, endocervix, ovary, pancreas, kidney, endometrium, rectum, colon, breast, and thyroid. However, the same trend was not found in a pooled squamous cell carcinoma (SCC) dataset [34], suggesting the gene is a potential driver of ADs and may act as a therapeutic target for cancers, particularly ADCs.

At present, at least two miRNAs have been identified to inhibit lung cancer development partly by targeting 3′-UTR of IGF2BP1. MiR-494 was found to be significantly enhanced in cell lines and serum of NSCLC patients as well as closely associated with poor clinical outcome [35, 36]. However, the upregulation of miR-494 was demonstrated to suppress colony-forming activity and cell proliferation, as well as induce senescence in A549 cells via directly downregulating IGF2BP1 levels and increasing the levels of IGF2BP1’s target IGF2 mRNA [37]. This means that the tumor-suppressive role of miR-494 by downregulating IGF2BP1 in lung cancer might be covered by other carcinogenic effects from elevating IGF2BP1 levels. MiR-491-5p, which may suppress the growth and metastasis of multiple types of tumors by targeting some cancer-related genes, was observed to be downregulated in NSCLC tissues and cell lines. In a mouse model, upregulation of miR-491-5p was observed to enhance the tumor cell cycle arrest at the G1/G0 stage and promote tumor cell apoptosis, as well as repress tumor cell proliferation, migration, and invasion, and growth by inhibiting IGF2BP1 [24].

### Liver cancer

IGF2BP1 expression has been reported in gallbladder cancer (GC), hepatocellular carcinoma (HCC), and fibrolamellar hepatocellular carcinoma (FL-HCC). In GC tissues, the positive expression of IGF2BP1 was 72.4%, with the lower expression levels compared to control tissues. Furthermore, IGF2BP1 expression was observed to be related to longer survival and better prognosis [38]. However, IGF2BP1 expression was found to promote HCC cell proliferation, migration, and invasion [39, 40], and correlate with poor survival and prognosis [41–43]. Those observations indicated the different roles of IGF2BP1 in survival and prognosis for distinct liver cancers.

In vitro, SOX12 upregulation was found to promote HCC cell growth and apoptosis, invasion, and metastasis partly via enhancing IGF2BP1 expression that elevates the expression of c-MYC and the proliferation marker MKI67 [3, 41, 44]. Some lncRNAs function in HCC progression by various mechanisms, such as splicing regulation and lncRNA-miRNA/protein interaction [45–47]. In previous reports, lncRNA HCG11 was showed to be a tumor suppressor in prostate cancer (PCa) [48] and may serve as prognostic markers in both breast and gastric cancers [49, 50]. However, in HCC development, HCG11 was demonstrated to be highly expressed and unregulated in the activity of IGF2BP1, by which it modulated the downstream signaling of IGF2BP1, including p21/capase-3 and MAPK, and subsequently affected the growth and metastasis of HCC. Nevertheless, knockdown of HCG11 was showed to lead to IGF2BP1 suppression and inhibition of cell viability and proliferation, cell migration and invasion, and colony formation ability of HepG2 cells, as well as induce cell apoptosis and cell cycle arrest at G1 stage, which is similar to the effects of IGF2BP1 suppression by shRNA [40]. Another lncRNA HULC was also found to be overexpressed in HCC tissues and correlate with low-grade and low-stage HCC, indicating a functional role of HULC in the early stages of tumor development. Interestingly, IGF2BP1 served as a trans-acting factor to inhibit HULC expression by recruiting CNOT1 and bringing HULC into close proximity to the CCR4-NOT deadenylase complex [51]. Significantly, in HCC samples, both IGF2BP1 and HULC were upregulated [51], suggesting that other modulation may antagonize the suppressive effects of IGF2BP1 on HULC.

Presently, some miRNAs including miR-625, miR-98-5p, miR-9, miR-1275, and miRNA-196b were showed to be frequently downregulated in HCC samples, and their upregulation may hinder HCC development [39, 42, 43, 52, 53]. miR-625 was found to bind to IGF2BP1 and inhibit tumor migration and invasion, which may have partly resulted from the speculated IGF2BP1/PTEN/HSP27 pathway in which the re-expression of miR-625 might indirectly reduce PTEN expression through depressing IGF2BP1, subsequently contributing to the Akt-mediated phosphorylation of HSP27 and suppressing F-actin polymerization [42]. miR-98-5p was reported to associate with poor survival of HCC patients and could depress HCC cell proliferation and induce cell apoptosis by targeting and inhibiting IGF2BP1 [39]. Another potential prognostic marker for HCC, miR-9, was observed to promote HCC cell proliferation and migration by hypermethylation-mediated downregulation of it and inhibit HCC development via suppressing AKT and ERK phosphorylation that are well known for their oncogenic properties after targeting IGF2BP1 [43]. miR-1275, prevalent frequently in various cancers, could hinder HCC cell growth partially by simultaneously regulating the oncogenic IGF2BP1–3 and IGF1R [52]. Furthermore, IGF2BP1 was found to be targeted by miRNA-196b and suppress cell proliferation and induce apoptosis in HepG2 cell [53].

### Breast cancer

IGF2BP1 was found to ubiquitously express in normal adult breast epithelial cells, and mouse and human breast tumor cell [1]. However, the suppression of invasion and metastasis by IGF2BP1 was seen in breast carcinoma cells of human and rat. IGF2BP1 activation may depress chemotaxis and metastasis of breast cancer cells through sustaining cell polarity and directional movement by modulating the localization of β-actin mRNA [54–56]. The high promoter methylation and significant downregulation of IGF2BP1 was observed in all metastatic cell lines including MTLn3, MDA435, MDA231, and 4T1 but slightly in non-metastatic cell lines including MTC in T47D, and the promoter demethylation of IGF2BP1 induced its endogenous expression in metastatic MTLn3 cells, indicating epigenetic modifications could act a role in silencing the IGF2BP1 in metastatic breast tumor cells [54, 57]. Furthermore, IGF2BP1 can depress metastatic breast tumor cell proliferation and invasion by targeting and regulating localized expression of multiple adhesion- and motility-related mRNAs. Repression of IGF2BP1 expression may reduce the accumulation of E-cadherin, a crucial cell adhesion protein, at cell–cell contacts, as well as impair the dynamics of focal adhesions, subsequently converting the polarized adherent phenotype into an unpolarized morphological one with invasive behavior [57]. Notably, promoter methylation of IGF2BP1 was not detected in normal tissues including the breast, liver, and brain of adult rat. Taken together, it seems possible to draw a conclusion from these in vitro and in vivo studies that methylation events of IGF2BP1 are becoming more frequent with the higher breast cancer grade, and this leads to more silence and downregulation events of IGF2BP1, resulting in dysregulated effects on IGF2BP1-target mRNAs. Significantly, in addition to methylation, other factors also cause silence event of IGF2BP1 [54]. However, in another observation of metastatic human breast tumor-driving cell and xenograft mouse model, the gain expression of IGF2BP1 inhibited tumor growth and metastases, which may be through the function of the KH3/4 domain of IGF2BP1 on its targeted mRNAs [17], suggesting other tumor-related inhibition paths for IGF2BP1 exist in addition to methylation-induced effects. For example, IGF2BP1 expression might upregulate RGS4 mRNA and inhibit tumor cell proliferation and invasion, while downregulate GDF15, IGF2, and PTG2 mRNAs and lead to suppression of tumor cell proliferation and invasion [17].

However, inconsistent with the suppressive role in the breast tumor cell-derived cell and xenograft mouse models, some in vivo studies revealed that IGF2BP1 plays an enhancive role in tumorigenesis and metastasis in breast cancer cells [57]. The probable explanations for contradictive function of IGF2BP1 might result from the cell-specific differences and the endogenous difference between non-metastatic cells and metastatic cells, for example, the significantly high promoter methylation of IGF2BP1 in metastatic breast tumor cells compared with that in non-metastatic breast tumor cells results in the more common silence events of IGF2BP1. In addition, in cancer cells advance to metastasis, crucial genes have incensed changes in the pattern of expression including both the gain and loss of gene function [58].

### Gynecologic cancers

IGF2BP1 was reported to be significantly elevated in cervical cancer (CC) tissues and cell lines and in ovarian cancer tissues [59–61]. Two miRNAs including miR-140-5p and miR-124-3p were significantly downregulated in CC tissues compared with that in normal cervical tissues and cell lines. In addition, CC patients with higher miR-140-5p levels had significantly longer survival, ectopic expression of miR-124-3p, and inhibited tumor cell growth and metastasis. Therefore, miR-140-5p and miR-124-3p might act as an inhibitor of cell proliferation, migration and invasion, growth, and metastasis in CC by targeting and downregulating IGF2BP1 levels [60].

In ovarian carcinoma (OC) cells, IGF2BP1 was showed to act as an oncogenic factor that contributes to enhanced proliferation by stabilizing the c-MYC mRNA. Furthermore, elevated IGF2BP1 expression was observed preferentially in high-grade and high-stage cases [61], indicating its prognostic role for decreased recurrence-free and overall survival. Additionally, IGF2BP1 was demonstrated to sustain tumor cell survival upon binding eIF5A and suppressing eIF5A-mediated apoptotic effects that depends on the cytoplasmic IGF2BP1 levels in ovarian and breast cancer of mouse [62], indicating their important role in the therapeutic trials using inhibitors of exportin1 (XPO1) that is the key nuclear export protein and essential for transporting cargo proteins with leucine-rich nuclear export sequences from the nucleus to the cytoplasm, such as leptomycin B [62, 63]. The let-7 miRNA family was dysregulated in various cancers, including OC, and had a tumor-suppressive role by interfering in the expression of multiple oncogenic factors, including IGF2BP1 [64, 65]. let-7 was demonstrated to inhibit tumor cell growth and migration, and self-renewal via depressing the oncogenic “triangle” composed of HMGA2-IGF2BP1-LIN28B [65]. Interestingly, another underexpressed miR-708 was observed to enhance the susceptibility of OC cells to cisplatin through targeting IGF2BP1 and inducing an inhibition of Akt signaling that plays a critical role in cisplatin resistance in OC. However, the upregulated expression of IGF2BP1 might restore resistance of miR-708-overexpressing OC cells to cisplatin [66].

### Gastrointestinal cancers

IGF2BP1 was demonstrated to play tumor-suppressive or tumorigenic roles in gastrointestinal cancers. Stromal IGF2BP1 is a contributor to normal intestinal development and homeostasis. Mongroo et al. have identified the tumorigenic role of IGF2BP1 in colon cancer cell. IGF2BP1 loss was found to inhibit the expression of K-Ras and Cdc34, the let-7 repressor Lin-28B, and c-Myc, concomitantly depress anchorage-independent growth and colon cancer cell proliferation, and trigger caspase-mediated cell death [67]. During the process, c-Myc and IGF2BP1 constitute a potential feedback mechanism to reciprocally regulate expression of each other in colon cancer. In the study, IGF2BP1 was established to promote colon cancer cell survival and regulate K-Ras expression via targeting 3′-UTR of K-Ras mRNA that frequently mutates in human cancers and regulates distinct cellular pathways important for the growth, differentiation, and survival of cell [68], in part by suppressing CYFIP2, which is a p53-inducible gene and may depress cell proliferation and caspase activation, and induce apoptosis in colon cancer [69]. However, different from the oncogenic role in previous colorectal tumors and cells, and other cancers [67, 70], IGF2BP1 was demonstrated to have tumor-suppressive roles. Under the stromal knockdown of IGF2BP1 in the mouse model of colitis-associated cancer, the mice showed elevated tumor burden such as enhanced tumor initiation and progression, which is partly explained by the effects of chronic inflammatory damage caused by IGF2BP1 detection [16]. Additionally, IGF2BP1 loss enhanced the levels of HGF, which is produced by stromal fibroblasts and contributes to epithelial cell proliferation and invasive growth of CRC cells by the interaction with β-catenin signaling, and conferred resistance to EGFR inhibitors in colon tumor-initiating cells in fibroblasts in vitro, and increased fibroblast cell growth [16, 71–74], indicating a potentially tumor-suppressive role of IGF2BP1 via modulating HGF in fibroblasts. The contradictory function of IGF2BP1 in colon cancer cells and mouse models of colitis-associated cancer may result from use of different tumor models or the complexity of colon cancer tumorigenesis, suggesting that tumor cells arrived from different origins or conditions might have different responses to IGF2BP1 expression.

LncRNA GHET1 was found to be upregulated in gastric carcinoma tissues and correlate with poor prognosis, with a positive relationship with tumor size and invasion, and the shorter survival. Enhanced GHET1 expression can contribute to gastric carcinoma cell proliferation and tumor growth in vitro and in vivo by physically associating with IGF2BP1 and increasing the physical interaction of IGF2BP1 with c-Myc mRNA, consequently enhancing c-Myc mRNA stability and expression [75].

### Sarcoma

Although the study of IGF2BP1 in osteosarcoma (OS) is little, the observation of the restoration of miR-150 expression in OS cells could suppress the proliferation, migration and invasion, and growth of tumor cell and induce apoptosis by targeting it was reported [76]. MiR-150, which has been demonstrated to play important roles in various human cancers, was showed to be downregulated in OS tissues and cell lines in contrast to the matched adjacent tissues as well as human normal osteoblast cells.

IGF2BP1 is a critical modulator of cellular inhibitor of apoptosis 1 (cIAP1) expression and of apoptotic resistance in rhabdomyosarcoma (RMS), controlling cell death and drug resistance by medicating translation of cIAP1. In rhabdomyosarcoma tissues and cell lines, IGF2BP1 was found to be overexpressed and drive the expression of cIAP1 that is a key modulator of apoptosis and contributes to tumor cell survival through controlling the nuclear factor-κB signalling and extrinsic cell death pathways, as well as promote disease progression and chemo-resistance by increasing IRES-mediated translation of cIAP1 [77, 78]. Decreasing levels of cIAP1 in RMS cell lines upon IGF2BP1 knockdown sensitize RMS cells to tumor necrosis factor-α (TNFα)-mediated cell death, similar to the result by Smac mimetic compound (SMC) treatment. Moreover, targeting cIAP1 by SMC suppresses the formation and growth of RMS xenograft tumors in mice [78].

### Central nervous system cancer

The important roles of IGF2BP1 in neuroblastoma, meningiomas, and glioblastoma were reported [79]. IGF2BP1 was showed to highly express in neuroblastoma tissues and identified as a significantly important gene in this disease because of its clear negative prognostic effect at the DNA, mRNA, and protein levels, and its positive correlation with MYCN, a most prominent oncogene in neuroblastoma and other aggressive tumors. In the 69 neuroblastoma tissues, IGF2BP1 DNA copy number, mRNA, and protein abundance were strikingly higher in stage 4 than stage 1 tumor. Additionally, IGF2BP1 mRNA and protein levels were correlated with poor overall survival. IGF2BP1 clearly influenced MYCN expression and neuroblastoma cell survival [80].

The significantly frequent promoter methylation of IGF2BP1 in recurrent meningioma cases was found [81]. IGF2BP1 increased the malignant potential of meningiomas, with significantly higher methylation levels in atypical (grade II/III) than benign (grade I), indicating its prognostic role. Notably, the median production of protein of this gene for both of the two-set atypical meningiomas was reduced, though without statistically significant difference [82]. Those findings show that methylation of IGF2BP1 involves tumor development and regulates its expression and thus modulates the downstream biological pathway.

Glioblastoma multiforme (GBM) is known as the most highly malignant and active primary brain tumor with a poor prognosis [44]. Some miRNAs including miR-506 and miR-873 were showed to be lowly expressed in GBM tissues or cells and play a tumor-suppressive role in this disease by targeting and modulating IGF2BP1. However, their overexpression was observed to repress the cell proliferation and migration, and invasion of GBM through downregulating the IGF2BP1 levels, which reduces the level of c-MYC, CD44, MKI67, and PTEN mRNA in GBM cells or blocks G1/S transition in glioblastoma cell [44, 83]. C-MYC and MKI67 act as effective modulators of cell proliferation and apoptosis with the stability by IGF2BP1 [41]. Furthermore, suppression of CD44 mRNA degradation may promote invadopodia formation under the IGF2BP1 upregulation [84]. PTEN has a cell-migration role in early neural precursors, and IGF2BP1 can enhance the directionality of cell migration with the functional PTEN-dependent manner [85, 86]. Those studies support a hypothesis that reduced levels of miR-506 and miR-873 or other miRNAs contribute to carcinogenesis and metastasis via upregulating IGF2BP1 expression and subsequently modulating its target RNA transcripts.

### Melanoma

Upregulation of IGF2BP1 expression in human melanoma or mouse model was observed [87, 88]. Its overexpression influences various proto-oncogenes and oncogenic signaling pathways that involve tumor development, survival, and drug resistance [88]. In metastatic melanoma, IGF2BP1 expression was observed to confer a resistance to chemotherapeutic agents, whereas its inhibition or knockdown enhanced the effects of chemotherapy and reduced tumorigenic characteristics [88]. Moreover, IGF2BP1 knockdown was found to reduce levels of c-MYC, which contributes to the suppression of NF-kB activity and of anchorage-independent growth of melanoma cells and proliferation, as well as induces apoptosis [89]. Thus, IGF2BP1 could be a reduce-chemoresistance target for melanoma.

### Leukemia

The role of IGF2BP1 in hematological malignancies, including acute myeloid leukemia (AML) and B acute lymphoblastic leukemia (ALL) was found [4], although its expression and exact function is little known. LIN28B, a stem cell reprogramming factor, may downregulate the let-7 family to promote stem cell differentiation [90, 91]. The overexpression of this gene is common in advance leukemias [92] and could depress cancer stem cell (CSC) differentiation [93–96], while its inhibition might induce G2/M cell-cycle arrest which regulates cell proliferation and colony formation in AML. Importantly, IGF2BP1 has been identified as a downstream effector of LIN28B by let-7 miRNA. By this mechanism, overexpression of LIN28B may inhibit let-7 miRNAs, thus elevating IGF2BP1 expression in AML cells [4]. Additionally, the loss of IGF2BP1 was indicated to increase tumor cell proliferation related to increased IGF-II protein levels in K562 leukemia cells [97] and promote PARP- and caspase-3 mediated apoptosis in colorectal cancer cells [67]. However, whether LIN28B also induces PARP- and caspase-3-mediated apoptosis in AML and other cancers by downregulating IGF2BP1 is unknown. In t(12;21) (p13;q22)-positive ALL, IGF2BP1 was found as a potent regulator of ETV6/RUNX1 mRNA stability and potentially linked this evolutionary highly conserved protein to cell transformation events [98].

### Other cancers

IGF2BP1 was showed to be overexpressed in both retinoblastoma and choriocarcinoma. In retinoblastoma, the gene promotes tumor cell proliferation and migration [99]. In choriocarcinoma cell lines, the depletion of ribosomal protein S6 kinase (RSK2) or protein phosphatase methylesterase 1 (PPME1) inhibits cell migration and invasion, which is similar to that after knockdown of IGF2BP1 that controls RSK2 and PPME expression, whereas it did not influence cellular proliferation and morphology [100], indicating that IGF2BP1 promotes choriocarcinoma cell migration and invasion partly via the effect of RSK2 and PPME1.

## The interconnectivity of IGF2BP1 with its targeted RNAs in cancers

**Fig. 2:**
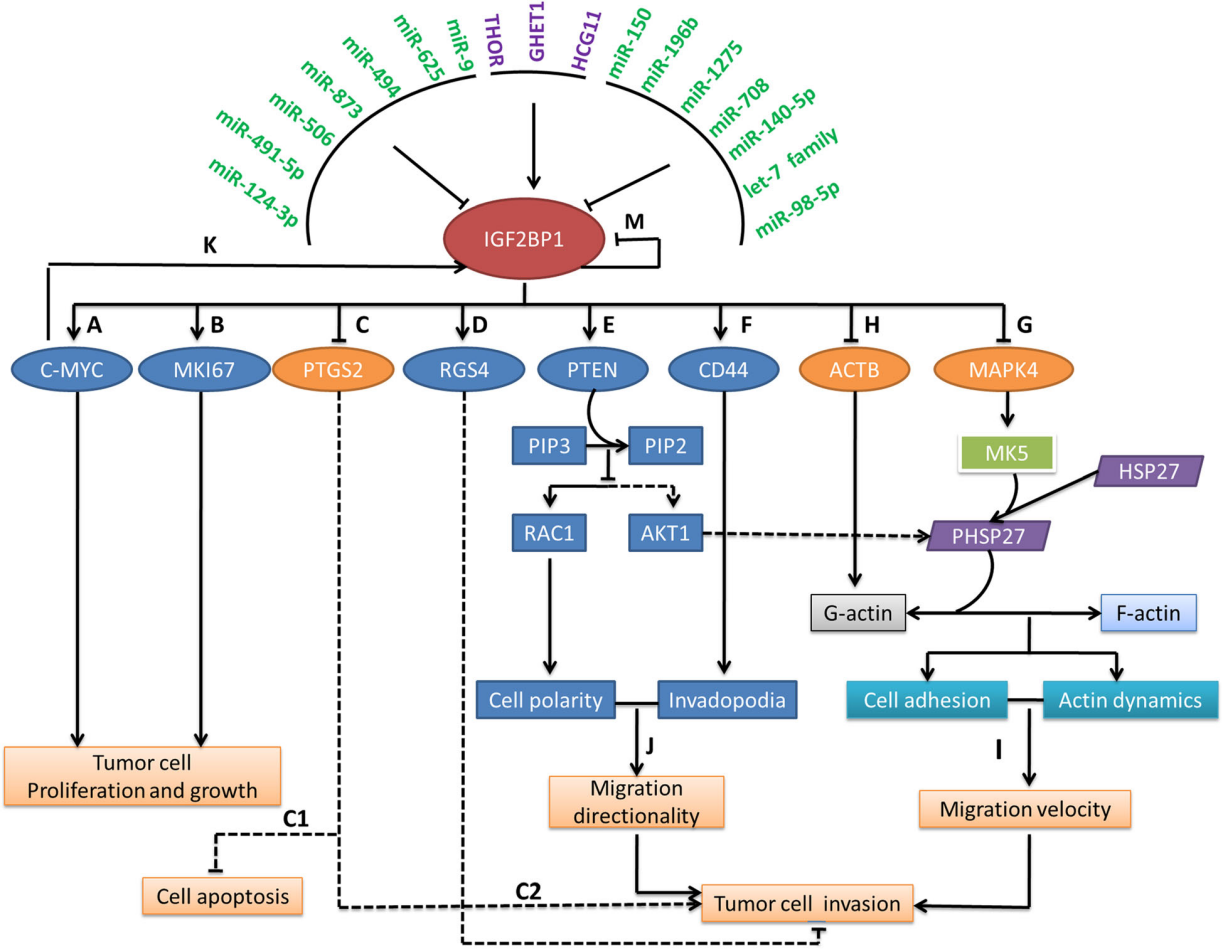
The roles of IGF2BP1 in promoting and suppressing tumor growth and invasion via regulating different mRNA targets, under the modulation of upstream non-coding RNAs. (1) Some miRNAs/lncRNAs upregulate or downregulate IGF2BP1 expression levels. (2) IGF2BP1 promotes the expression of c-MYC and MKI67 by stabilizing their transcripts and promotes tumor cell proliferation and growth (**a**, **b**). In addition, IGF2BP1 elevates CD44 and PTEN expression via preventing mRNA turnover. The enhancement of CD44 expression induces the formation of invadopodia and therefore may promote the tumor cell migration and invasiveness (**f**, **j**). Elevation of PTEN inhibits PIP3/PIP2 ratios and then interferes with the activation of RAC1, which enhances cell polarization and thus contributes to directed tumor cell migration as well as tumor invasion (**e**, **j**). IGF2BP1 suppresses the expression of MAPK4 and ACTB through interfering with mRNA translation (**g**, **h**). The inhibition of MAPK4 antagonizes MK5-directed phosphorylation of HSP27. PHSP27 at both residues induces the degradation of oligomers and increases the sequestering of actin monomers by the phosphorylated protein. The reduced ACTB also decreases G-actin levels. This shifts the cellular G-/F-actin equilibrium contributes to cell adhesion and actin dynamics and finally promotes cell migration velocity (I) [41, 84–86, 101, 102]. Furthermore, IGF2BP1 promotes RGS4 expression and thus indirectly depresses tumor cell invasion (**d**). IGF2BP1 inhibits PTGS2 expression (**c**). The reduction of PTGS2 indirectly promotes tumor cell invasion and releases the suppression for cell apoptosis (C1, C2) [17]. C-Myc and IGF2BP1 constitute a potential feedback mechanism to reciprocally regulate expression of each other (**k**) [68]. The hypermethylation of promoter in IGF2BP1 leads to its expression silencing (**m**) [81, 82].The gray dotted lines show that the interaction of the depicted pathways needs to be explored. The part of Fig. 2 including pathway from (**e**, **f**, **h**, and **g** to **i** and **j** is adapted from the Figure 3 of the paper by Stohr et al. [102]

IGF2BP1 was found to be commonly and significantly upregulated in almost all tumor cell lines and tumor tissues with a range from 17 to 72.4% of incidence **(**Table 1**)** and act as an essential oncogene that promotes the stability, localization, and translation of cancer-related mRNA targets by the regulation of some lncRNAs and miRNAs (Table 2). Almost all of the reported miRNAs including miR-491-5p, miR-625, miR-98-5p, miR-9, miR-196b, miR-1275, miR-708, let-7 miRNA family, miR-150, miR-506, miR-873, miR-140-5p, and miR-124-3p are downregulated in corresponding cancer samples, whereas all of the three lncRNAs including HCG11, GHET1, and THOR are highly expressed in corresponding solid tumors. As shown in Fig. 2, upregulation of the three lncRNAs can elevate IGF2BP1 level, while upregulation of the miRNAs could repress IGF2BP1 expression. HCG11 can upregulate the activity of IGF2BP1 and thus leads to activation of MAPK signaling [40]. GHET1 may enhance he stability of c-Myc mRNA and expression by physically associating with IGF2BP1 and elevating the physical interaction between c-Myc mRNA and IGF2BP1 [75]. However, THOR indirectly controls the levels of IGF2BP1-target mRNAs including KRAS, IGF2, CD44, PABPC1, ACTB, GLI1, MYC, CTNNB1, MAPT, PPP1R9B, PTEN, BTRC, and H19. Nearly the levels of all those targets are reduced in both MM603 and H1299 cells with THOR knock down similar to that with IGF2BP1 knockdown, whereas elevates with overexpression of THOR in SKMEL5 and H1437 cells [29]. Taken together, those findings show that lncRNAs HCG11, GHET1, and THOR dysregulate the expression of IGF2BP1-downstream targets by modulating IGF2BP1. Similarly, the miRNAs in tumors target the 3′-UTR of IGF2BP1, by which they regulate the expression of IGF2BP1-downstream targets and affect tumor cell proliferation, migration and invasion, and growth.

**Table 1:** The expression and roles of IGF2BP1 in human cancer

Cancer type	Incidence	Method	The status of expression in tissues	The roles of IGF2BP1 expression in cancers	Reference
Lung cancer and esophageal adenocarcinomas					
Non-small cell lung cancer	27% (4/11)–52% (139/267)	Microarray, qRT-PCR, Western blot	Overexpressed	Promoting lung cancer development and progression	[8, 33]
Lung adenocarcinoma	17% (1/6)	IHC, TCGA datasets	Significantly upregulated	Poor overall survival; bigger primary tumor size	[32, 34, 79]
Lung squamous cell carcinoma	37.5% (3/8)	IHC, TCGA datasets	Significantly upregulated	Younger age at diagnosis	[32, 79]
Esophageal adenocarcinomas	Unknown	TCGA datasets	Significantly upregulated	Poor survival	[34]
Breast cancer					
Breast cancer	59% (59/118)	IHC, immunofluorescence, Western Blotting, RNASeq	High and ubiquitously expressed	Tumorigenic activity, clonogenic growth	[1, 8]
Breast cancer		qRT-PCR	Wildly expressed	Inhibiting tumor growth and metastasis	[17, 54–57]
Liver cancer					
Gallbladder carcinoma	*72.4%*	IHC	Ubiquitously expressed but lower expressed than controls	Longer survival	[38]
Hepatocellular carcinoma	Unknown	TCGA, IHC, qRT-PCR, Western blot		Poor survival	[3]
Hepatocellular carcinoma		qRT-PCR, Western blot	Overexpressed	Positively correlated with tumor size, advanced stages and grading of poor differentiation	[41]
Hepatocellular carcinoma		IHC	Significantly upregulated	Promoting progression; migration and invasion	[40]
Hepatocellular carcinoma		IHC	Significantly upregulated	Poor prognosis	[42]
Hepatocellular carcinoma		qRT-PCR, Western blot	Significantly upregulated	Promoting tumor cell proliferation, migration, and invasion	[39]
Hepatocellular carcinoma		ELISA, qRT-PCR, Western blot	Significantly upregulated	Poor post-surgery prognosis	[43]
Hepatocellular carcinoma		qRT-PCR	Significantly upregulated		[52]
Fibrolamellar hepatocellular carcinoma (FL-HCC)	Unknown	ACGH, RNA-seq	Significantly upregulated		[50]
Leukemia					
Chronic myelogenous leukemia (CML)	Unknown	qRT-PCR	Significantly upregulated	Promoting cell proliferation	[97]
Acute lymphoblastic leukemia (ALL)	Unknown		Overexpressed		[98]
Melanoma					
Melanoma	34% (13/38)	RT-PCR	Upregulated	Promoting tumor growth and drug resistance	[8, 87–89]
Osteosarcoma (OS)					
Osteosarcoma (OS)	Unknown	Western blot	High expression	Promoting the proliferation, migration and invasion, and growth of tumor cell, and inhibiting apoptosis	[76]
Gastrointestinal cancer					
Colon	50% (36/79)–59% (46/78)	IHC		Tumor-suppressive role	[8, 16]
Colon		qRT-PCR, immunofluorescence, IHC, Western Blotting	Upregulated	Promoting cell proliferation, growth, and survival	[67]
CNS cancer					
Neuroblastoma	Unknown	Microarray data	High expression	Poor survival, advanced stages	[80]
Glioblastoma multiforme (GBM)	54.5% (6/11)	qRT-PCR, Western blotting	Significantly upregulated	Inducing carcinogenesis and metastasis	[44, 79]
Meningiomas	63.6% (14/22)	Western blotting	Reduced expression	Promoting tumor development and the malignant potential	[81, 79]
Rhabdomyosarcomas (RMS)	Unknown	IHC	Overexpressed tumors and cell lines	Regulating cell death and drug resistance	[78]
Gynecologic cancers					
Cervical cancer (CC)	Unknown	Western blotting, qRT-PCR	Significantly upregulated	Promoting cell viability, migration, and invasion	[59, 60]
Ovarian cancer	69% (73/106)	IHC	High expression	Poor prognosis and poor overall survival	[8, 61]
Other cancers					
Choriocarcinoma (CC)	Unknown	IHC, Western blotting, qRT-PCR	Overexpressed	Promoting cell migration and invasion	[100]
Retinoblastoma	Unknown	LC–MS/MS,IHC	Overexpressed	Promoting cell proliferation and migration	[99]

**Table 2:** Target mRNAs of IGF2BP1

Identified/putative target mRNAs	Cis-element on RNA	Regulation of IGF2BP1 for target mRNA	The biological roles of those target mRNAs (http://www.genecards.org/)	Ref.
eEF2		Increasing basal protein translation rates	Enhancing basal proliferation rates	[102]
CD44	3′-UTR	Stabilization of the transcript	Involving in invadopodia formation, cell migration, tumor growth and progression.	[83]
c-Myc	CDS	Inhibition of CRD-dependent mRNA decay	Promoting the tumor cell proliferation	[40]
ACTB	3′-UTR	Inhibition of mRNA translation;	Involving in various types of cell motility	[101]
IGF2	5′-UTR	Inhibition of mRNA translation	Promoting cell growth and proliferation	[8]
PTEN	CDS	Inhibition of CRD-dependent mRNA decay	Modulation of actin dynamics and cell migration; modulating cell cycle progression and cell survival; promoting cell polarization and directed movement.	[100]
MDR1	CDS	Inhibition of CRD-dependent mRNA decay	NA	[8]
MAPK4	3′-UTR	Inhibition of mRNA translation	Modulating actin dynamics and cell migration; promoting entry in the cell cycle	[100]
PPP1R9B	3′-UTR	mRNA transport	Involving in linking the actin cytoskeleton to the plasma membrane at the synaptic junction	[8]
BTRC	CDS	Inhibition of betaTrCP1 mRNA degradation	Mediating the ubiquitination and subsequent proteasomal degradation of target proteins	[102]
CTNNB1	3′-UTR	Inhibition of mRNA decay	Regulating cell adhesion; promoting neurogenesis	[8]
KRAS	CDS, 3′-UTR	Inhibition of mRNA decay	Modulating cell proliferation; promoting oncogenic events	[66, 67]
PABPC1	5′-UTR	mRNA translation	Regulating mRNA metabolism, and translationally coupled mRNA turnover	[29]
H19	3′-UTR	mRNA localization	Involving in migration and invasion	[29]
GLI1	CDS	Inhibition of mRNA decay	Regulating cell proliferation and differentiation; promoting cancer cell migration	[29]
cIAP1	5′-UTR IRES	Enhancing IRES-mediated translation	Modulating cell proliferation, as well as cell invasion and metastasis, and cell cycle	[77)
RAPGEF2			Inhibiting cell proliferation of melanoma cells and promotes their apoptosis; regulating embryonic blood vessel formation; establishment of basal junction integrity and endothelial barrier function	[100]
RPS6KA5			Involving in neuronal cell death; limiting the production of pro-inflammatory cytokines	[100]
RSK2		Stabilization of the transcript	Promoting cell migration and invasion	[99]
PPME1		Stabilization of the transcript	Promoting cell migration and invasion	[99]
ITGA6	NA	NA	Mediating cell adhesion to extra cellular matrix or to other cells, and fertilization of ova and embryonic development	[28]
ETV6/RUNX1 mRNA (potential)		Stability of this transcript	NA	[97]
GDF15	3′-UTR	Inhibition of this transcript	Inhibiting breast cancer cell migration and invasion	[17]
RGS4			Inhibiting signal transduction and breast cancer cell migration and invasion	[17]
PTGS2			Modulating production of inflammatory prostaglandins, and motility, proliferation, and resistance to apoptosis.	[17]
CDH1		Localization of the mRNAs	Regulating cell–cell adhesions, mobility, and proliferation of epithelial cells; downregulation of cell growth	[53]
β-actin		Localization of the mRNAs	Involving in establishment of cell polarity and cell motility	[53]
α-actinin		Localization of the mRNAs	Regulating focal adhesion metabolism	[53]
Arp2/3		Localization of the mRNAs	Regulating focal adhesion metabolism, actin polymerization, and the formation of branched actin networks	[53]
TAU	3′-UTR	Localization of the mRNAs	Promoting microtubule assembly and stability; establishment and maintenance of neuronal polarity	[53]
MKI67		Stability of this transcript	Promoting the tumor cell proliferation	[40]

In the context of modulation by miRNAs/lncRNAs (Fig. 2), aberrant upregulation of IGF2BP1 promotes the expression of c-MYC and MKI67, as well as CD44 and PTEN [41, 44, 84, 101]. By stabilizing c-MYC and MKI67 transcripts, IGF2BP1 enhances tumor cell proliferation and growth. Interestingly, c-Myc and IGF2BP1 each constitutes a potential feedback mechanism to reciprocally regulate expression of the other. Additionally, IGF2BP1 prevents CD44 and PTEN mRNA turnover, consequently enhances CD44 expression, and induces the formation of invadopodia and therefore may promote tumor cell migration and invasiveness [84]. The elevation of PTEN inhibits PIP3/PIP2 ratios and then interferes with the activation of RAC1, which enhances cell polarization, and thus, this contributes to directed tumor cell migration as well as tumor invasion. However, the increased RGS4 expression enhanced by IGF2BP1 depresses tumor cell migration and invasion [17]. Although the expression levels of PTGS2, ACTB, and MAPK4 are reduced by IGF2BP1 interfering with their mRNA translation, there are different results on tumor events. Reduced PTGS2 decreases the suppression of tumor cell apoptosis and the promotion of tumor cell invasion [17]. The inhibition of MAPK4 antagonizes MK5-directed phosphorylation of HSP27. PHSP27 at both residues induces the degradation of oligomers and increases the sequestering of actin monomers by the phosphorylated protein [101]. The reduced ACTB also decreases G-actin levels. This shift in the cellular G-/F-actin equilibrium contributes to cell adhesion and actin dynamics, and finally promotes cell migration velocity [102]. In addition, promoter methylation of IGF2BP1 may silence its expression and subsequently modulates the downstream biological pathways [81, 82]. Taken together, we can deduce a conclusion that IGF2BP1 plays an important role in the occurrence of tumor events and shows a degree of tumor suppressor effect. However, determining whether the occurrence of tumor events or not partly depends on IGF2BP1 targeting different cancer-related mRNAs, although the driving factors that contribute to this gene to choose different mRNA targets are unclear. It is worth noting that the methylation events of the promoter in IGF2BP1 at least involve the selected process because high promoter methylation of IGF2BP1 was demonstrated to block β-catenin binding to the IGF2BP promoter, leading to inactivation of the gene and enhancing proliferation and migration of metastatic breast cancer cells (Fig. 1d) [54, 103]. Additionally, the selective manner may partially result from the competitive combination on m^6^A-containing mRNAs between IGF2BP1 and other m^6^A-binding proteins such as YTHDFs, since they could read the same m^6^A regions of mRNAs while determining a totally different fate of m^6^A-containing mRNAs (Fig. 1c).

As per the tumor-promotion role of IGF2BP1 in most of cancers, it seems that IGF2BP1 and its targeted transcripts could be attractive anticancer drug targets; however, small molecule inhibitors of IGF2BP1 and other cancer-related mRNA stabilizing proteins, as well as the upstream ncRNAs, are little known [8]. Note worthily, a small molecule, BTYNB, might function as a potential therapeutics by inhibiting cell proliferation of IGF2BP1-positive cancer cells without effect in IGF2BP1-negative cells. BTYNB may restrain binding of IGF2BP1 to the coding region stability determinant of c-Myc mRNA and downregulate several mRNA transcripts including c-Myc, β-TrCP1, and eEF2 both in IGROV-1 and SK-MEL2 cancer cells, as well as decrease activation of nuclear transcriptional factors-kappa B (NF-κB). Moreover, it also selectively reduces the levels of other cancer-related IGF2BP1 mRNA targets including CALM1, CDC34, COL5A, and BTRC, similar to the effect by RNAi knockdown of IGF2BP1 both in IGROV-1 and SK-MEL2 cancer cells [104].

## Conclusions

IGF2BP1 is broadly and highly expressed in embryonic and tumor tissues. The bulk of correlative studies describing enhanced expression or de novo synthesis of IGF2BP1 in human cancer and animal model provide strong evidence that IGF2BP1 serves important roles in controlling embryonic development, as well as functions as an oncogenic factor in most of cancers. In most of the cancers, IGF2BP1 enhances tumor cell proliferation, survival, adhesion-independent growth and invasion, and chemo-resistance. The upregulated expression of IGF2BP1 is associated with poor overall survival and metastasis in multiple cancers. However, the tumor-suppressive role of IGF2BP1 has been observed in breast cancer and colon stromal cells. At least in breast cancer, it is confirmed that IGF2BP1 inhibits tumor cell growth and invasion. IGF2BP1 has both tumor-driving and tumor-suppressive roles in cancers in a context-based manner.

Although the origin of difference between oncogenic and tumor-suppressive roles is unclear, the apparently contradictory functions of IGF2BP1 may result from tumor cells arrived from different origins or conditions with different responses to IGF2BP1 expression. In this context, it seems that some undefined driver factors contribute to IGF2BP1 selectively binding and regulating its mRNA targets and lead to either development or suppression for tumors. Though the mechanism of IGF2BP1 selectively binding to its mRNA targets is unclear, epigenetic modifications of IGF2BP1 at least are involved in the process. In addition, the selective manner may partially result from the competitive combination on m^6^A-containing mRNAs between IGF2BP1 and other m^6^A-binding proteins such as YTHDFs.

The reasonable inferences about the functions of IGF2BP1 in cancer biology can be confirmed in the future by in vivo and in vitro studies, according to specific origins or conditions of the tumor cells. Additionally, the emerging miRNAs and lncRNAs that target and regulate IGF2BP1 have been ascertained to involve IGF2BP1-mediated mRNA modulation. Therefore, more interconnectivity between IGF2BP1 and its targeted mRNAs or ncRNAs can be explored in contexts related to specific cancer conditions. NcRNA-IGF2BP1-mRNA target-axes may be worth of more studies as their target potential for cancer therapy. Though currently available inhibitors of ncRNA-IGF2BP1-mRNA target-axes are limited, an inhibitor of IGF2BP1 binding to targeted mRNAs, BTYNB, has showed therapeutic potential by inhibiting cell proliferation of IGF2BP1-positive cancer cells.

## Abbreviations

CNC: Cranial neural crest
ELAVL1: ELAV-like RNA binding protein 1
IGF2BP1: Insulin-like growth factor 2 mRNA-binding protein 1 (IGF2BP1), also known as IMP/CRD-BP/VICKZ
lncRNA: Long non-coding RNA
miRNA: MicroRNA
ncRNAs: Non-coding RNA
RRMs: RNA recognition motifs
YTHDFs: YT521-B homology (YTH) domain-containing proteins
